# Correction: Impact of productive social safety net on households’ vulnerability to poverty in Tanzania

**DOI:** 10.1371/journal.pone.0323946

**Published:** 2025-05-27

**Authors:** Basil Msuha, Luitfred D. Kissoly

There is an error in affiliation 2 for authors Basil Msuha and Luitfred D. Kissoly. The correct affiliation 2 is: Department of Economics and Social Studies, School of Spatial Planning and Social Sciences, Ardhi University, Dar es Salaam, Tanzania.

## References

[1] Msuha B, Kissoly LD. Impact of productive social safety net on households’ vulnerability to poverty in Tanzania. PLoS One. 2024;19(8):e0308740. doi: 10.1371/journal.pone.0308740 39163303 PMC11335123

