# Supplementary material for: Impact of productive social safety net on households’ vulnerability to poverty in Tanzania
Source: PLoS One. 2024 Aug 20;19(8):e0308740. doi: 10.1371/journal.pone.0308740 (PMC11335123; doi:10.1371/journal.pone.0308740)
Supplement: S1 Appendix — (DOCX) [file pone.0308740.s001.docx]

**Appendix 1: Probit model for obtaining predicted probability of PSSN**

| **PSSN** | **Coefficient** | **Linearized std. err.** | **t** | **P>t** |
| --- | --- | --- | --- | --- |
| Eligibility | 0.102 | 0.007 | 14.92 | 0.000 |
| aefd | 0.000 | 0.000 | -64.14 | 0.000 |
| Age (years) | 0.005 | 0.001 | 6.6 | 0.000 |
| Age squared | 0.000 | 0.000 | 14.06 | 0.000 |
| Sex (1=male) | -0.232 | 0.004 | -58.35 | 0.000 |
| HH size (number) | 0.045 | 0.002 | 22.55 | 0.000 |
| HH size squared | -0.003 | 0.000 | -20.73 | 0.000 |
| Dependence ratio (ratio) | 0.007 | 0.001 | 5.65 | 0.000 |
| Marital status (1=No) | -0.340 | 0.005 | -71.7 | 0.000 |
| Location (1=rural) | 0.202 | 0.006 | 31.57 | 0.000 |
| Livestock (1=yes) | 0.039 | 0.004 | 10.28 | 0.000 |
| Employment (1=yes) | -0.148 | 0.004 | -35.25 | 0.000 |
| Remittances (1=yes) | 0.061 | 0.006 | 10.82 | 0.000 |
| Income sources (1=yes) | -0.404 | 0.009 | -46.59 | 0.000 |
| Food assistance (1=yes) | -0.111 | 0.020 | -5.47 | 0.000 |
| Health subsidy (1=yes) | 0.470 | 0.009 | 50.63 | 0.000 |
| Business ownership (1=yes) | 0.052 | 0.005 | 10.24 | 0.000 |
| Cons. | -1.388 | 0.025 | -54.53 | 0.000 |
| Number of strata: | 51 | Number of obs.: |  | 9,462 |
| Number of PSUs: | 796 | F(17, 729): |  | 5,433.82 |
| Population size: | 52,677,889 | Prob > F: |  | 0.000 |
| Design df: | 745 |  |  |  |

The predicted probability of PSSN obtained here was used as the instrumental variable (IV) for the treatment (PSSN) in two-stage least squares (2SLS).
