# Supplementary material for: Impact of productive social safety net on households’ vulnerability to poverty in Tanzania
Source: PLoS One. 2024 Aug 20;19(8):e0308740. doi: 10.1371/journal.pone.0308740 (PMC11335123; doi:10.1371/journal.pone.0308740)
Supplement: S2 Appendix — (DOCX) [file pone.0308740.s002.docx]

**Appendix 2:** **Complete IV regression results on the impact of PSSN on households’ vulnerability to poverty**

|  | **PSSN** | | **CCTs** | | **CCT+PWs** | |
| --- | --- | --- | --- | --- | --- | --- |
| **VP** | **First stage** | **Second stage** | **First stage** | **Second stage** | **First stage** | **Second stage** |
| Treatment |  | -0.134***  (0.032) |  | -0.140***  (0.034) |  | -0.389***  (0.121) |
| Age (years) | 0.000  (0.001) | 0.000  (0.000) | 0.000  (0.001) | 0.000  (0.000) | 0.002***  0.001) | 0.000  (0.000) |
| Age squared | 0.000  (0.000) | 0.000***  (0.000) | 0.000  (0.000) | 0.000***  (0.000) | 0.000**  (0.000) | 0.000**  (0.000) |
| Sex (1=male) | -0.016*  (0.009) | -0.013***  (0.002) | -0.018*  (0.009) | -0.013***  (0.002) | -0.001  (0.005) | -0.011***  (0.003) |
| HH size (number) | 0.002  (0.004) | 0.045***  (0.001) | 0.001  (0.004) | 0.045***  (0.001) | -0.002  (0.002) | 0.044***  (0.001) |
| HH size squared | 0.000  (0.000) | 0.000***  (0.000) | 0.000  (0.000) | 0.000***  (0.000) | 0.000  (0.000) | 0.000***  (0.000) |
| Dependence ratio (ratio) | 0.003  (0.003) | 0.044***  (0.001) | 0.003  (0.003) | 0.044***  (0.001) | 0.002  (0.002) | 0.044***  (0.001) |
| Marital status (1=No) | 0.000  (0.010) | -0.006**  (0.002) | -0.001  (0.010) | -0.006**  (0.002) | 0.001  (0.006) | -0.005*  (0.003) |
| Location (1=rural) | 0.021***  (0.007) | 0.113***  (0.002) | 0.018**  (0.007) | 0.113***  (0.002) | 0.012***  (0.004) | 0.115***  (0.003) |
| Livestock (1=yes) | 0.008  (0.006) | -0.067***  (0.001) | 0.005  (0.006) | -0.067***  (0.001) | -0.008**  (0.004) | -0.071***  (0.002) |
| Employment (1=yes) | -0.002  (0.007) | -0.003*  (0.002) | -0.001  (0.007) | -0.003  (0.002) | 0.007  (0.004) | 0.000  (0.002) |
| Remittances (1=yes) | -0.005  (0.009) | -0.003  (0.002) | -0.007  (0.009) | -0.003  (0.002) | -0.008  (0.005) | -0.005**  (0.003) |
| Income sources (1=yes) | 0.019  (0.016) | -0.107***  (0.003) | 0.011  (0.016) | -0.107***  (0.004) | 0.006  (0.010) | -0.107***  (0.005) |
| Food assistance (1=yes) | 0.004  (0.044) | -0.097***  (0.010) | -0.014  (0.043) | -0.100***  (0.010) | 0.004  (0.026) | -0.096***  (0.013) |
| Health subsidy (1=yes) | 0.068***  (0.024) | 0.001  (0.007) | 0.075***  (0.023) | 0.002  (0.007) | 0.001  (0.014) | -0.008  (0.007) |
| Business ownership (1=yes) | -0.004  (0.007) | -0.020***  (0.002) | -0.010  (0.007) | -0.021***  (0.002) | -0.002  (0.004) | -0.020***  (0.002) |
| Instrument (Z) | 0.647***  (0.094) |  | 0.622***  (0.092) |  | 0.224***  (0.055) |  |
| Cons | -0.017  (0.029) | -0.067***  (0.006) | -0.014  (0.028) | -0.066***  (0.006) | -0.042**  (0.017) | -0.081***  (0.008) |
| R^2^ | 0.068 | 0.8442 | 0.071 | 0.842 | 0.015 | 0.742 |

***P< 0.01; **P< 0.05, *P< 0.1; Figure in parenthesis are standard errors
