# Supplementary material for: Impact of productive social safety net on households’ vulnerability to poverty in Tanzania
Source: PLoS One. 2024 Aug 20;19(8):e0308740. doi: 10.1371/journal.pone.0308740 (PMC11335123; doi:10.1371/journal.pone.0308740)
Supplement: S3 Appendix — (DOCX) [file pone.0308740.s003.docx]

**APPENDIX 3: Postestimation: First-stage F-statistics and Durbin-Wu-Hausman (DWH) specification tests**

| **Ho: Instruments are weak** | | | **Ho: Variables are exogenous** | | | |
| --- | --- | --- | --- | --- | --- | --- |
| **First-stage F-statistics** | | | **Durbin-Wu-Hausman (DWH) specification tests** | | | |
| **Treatment Variable** | **F-statistic** | ***P*-Value** | **Durbin χ2 statistic** | ***P*-Value** | **Wu-Hausman *F* statistic** | ***P*-Value** |
| PSSN | 46.89 | <0.001 | 24.67 | <0.001 | 24.68 | <0.001 |
| CCTs | 45.67 | <0.001 | 24.84 | <0.001 | 24.86 | <0.001 |
| PWs | 16.68 | <0.001 | 25.54 | <0.001 | 25.56 | <0.001 |

Note: If the *F*-statistic is less than 10, the instrument is deemed weak as a rule of thumb.
