# Supplementary material for: Impact of productive social safety net on households’ vulnerability to poverty in Tanzania
Source: PLoS One. 2024 Aug 20;19(8):e0308740. doi: 10.1371/journal.pone.0308740 (PMC11335123; doi:10.1371/journal.pone.0308740)
Supplement: Attachment — Submitted filename: Response to Reviewers.docx [file pone.0308740.s006.docx]

**Impact of Productive Social Safety Net on Households’ Vulnerability to Poverty in Tanzania**

**PLOS ONE [PONE-D-24-02693]**

—Response to Reviewers—

Dear Muhammad Khalid Bashir, PhD

Thank you for giving us the opportunity to submit a revised draft of our manuscript titled *“****Impact of Productive Social Safety Net on Households’ Vulnerability to Poverty in Tanzania”*** to PLOS ONE. We appreciate the time and effort that you and the reviewers have dedicated to providing valuable feedback on our manuscript. We are grateful to the reviewers for their insightful comments regarding our manuscript. We have incorporated comments that reflect most of the suggestions provided. We have highlighted the changes made to the original version with tracked changes. This is a point-by-point response to the reviewers’ and editor’s comments and concerns.

**Response to Reviewer (Reviewer #1)**

Reviewer #1: This study empirically investigates the effect of SSNs on household’s vulnerability to poverty in Tanzania. To further improve the quality of the manuscript, the following suggestions are proposed:

1. The reviewed literature does not critically analyze the existing literature on (vulnerability to) poverty or on SSNs-Poverty nexus

**Response:** *Thank you for your feedback. In our revised manuscript, we have highlighted that previous research has mostly focused on evaluating the impact of SSNs on current poverty levels, with insufficient evidence available regarding their impact on future poverty, which deserves equal attention. In line with the comment, we have revised the literature by enhancing the introduction section and include a dedicated, separate section (section 2) for more empirical studies on SSNs-Poverty nexus*

2. The study does not provide suggestions for future research

**Response**: *Thank you for your suggestion. In the revised manuscript, we have incorporated Section 6, which includes a paragraph that provides additional information on the limitations of the study and identifies areas for future research.*

3. The figures, given in the end, are blurred (not clear)

**Response**: *Thank you for your observation. In the revised version of our manuscript, we have now retained only one figure (Figure 1. Conceptual framework for evaluating impact of SSNs) in print quality (600 dpi greyscale) and saved it in .jpeg format.*

*********************************************************************

**Response to Reviewer (Reviewer #2)**

Reviewer #2: the study on Impact of Productive Social Safety Net on Households’ Vulnerability to Poverty in Tanzania is very informative and healthy exercise for the readers on the poverty reduction topic particularly. The paper is scientifically sound and organized in proper manners. I have some suggestions for the authors:

1) The introduction part is more concise please elaborate it little bit more and clear the research gap of the study.

**Response:** *Thank you for this very important observation. In the revised version of the manuscript, we have explicitly conveyed the study's contributions to existing knowledge. We have also provided further elaboration in section 2*

2) The literature review part of the paper should also be enhanced. It is too small.

**Response**: *Thank you for your valuable feedback. In our revised manuscript, we have made substantial enhancements. Specifically, we have expanded Section 1 to clearly identify research gaps, and we have introduced a new section (Section 2) which explores the fundamental premise of social safety nets and delves into the conceptual framework that connects social safety nets to vulnerability to poverty. This section also explores empirical studies on this topic and identifies existing research gaps.*

3) The discussion part need more citations and references as well and discuss each variable impact on poverty vulnerability.

**Response**: *We appreciate your insightful observation. In section five (5. Discussions) of our revised manuscript, we have expanded our discussion according to the comments we received.*

4) provide the suggestion on the basis of the findings that authors found in the study not to be generalized but specific policy implications.

**Response**: *We are grateful for your input. In accordance with your suggestion, the revised manuscript now presents explicit policy implications that have been derived from the study's conclusions.*

**************************************************
